# A high-resolution genomic roadmap for chronic pain converges on glutamatergic neurons and C-fibers

**DOI:** 10.1172/JCI200554

**Published:** 2025-12-15

**Authors:** Erick J. Rodríguez-Palma, Rajesh Khanna

**Affiliations:** 1 Department of Pharmacology and Therapeutics and Center for Advanced Pain Therapeutics and Research (CAPToR), and; 2 McKnight Brain Institute, University of Florida College of Medicine, Gainesville, Florida, USA.

## Abstract

Chronic pain etiology involves a shared genetic profile, but its cellular context is poorly defined. In a study published in this issue of the *JCI*, Toikumo et al. integrated a chronic pain GWAS meta-analysis (*n* >1.2 million) with single-cell omics data from human brain and dorsal root ganglia. Genetic risk was predominantly enriched in central glutamatergic neurons, particularly those in the prefrontal cortex, hippocampus, and amygdala. In the periphery, the C-fiber nociceptor subtype hPEP.TRPV1/A1.2 was highlighted. Implicated genes converged on involvement in synaptic function and neuron projection development. This work identifies specific central and peripheral cell types that define the genetic architecture of chronic pain, providing a foundation for targeted translational research.

## Introduction

Chronic pain is not just a symptom, but also a disease that affects approximately 20% of the global population (1, 2). Despite its massive impact on health, most current treatments remain largely nonspecific, providing partial relief and often carrying the risk of addiction (3, 4). A key obstacle in developing better therapies has been identifying the provenance of pain — specifically, determining where in the body and in which cells its genetic roots lie. Large-scale studies that scan the entire human genome, known as GWAS, have revealed hundreds of genetic regions linked to chronic pain (5–7), which could help to accelerate drug discovery, resulting in higher drug approval rates. However, these findings have often been too broad to translate into specific biological targets for therapy.

In this issue of the *JCI*, Toikumo and colleagues bring much-needed clarity to this problem (8). By combining genetic data from more than 1.2 million people with high-resolution single-cell maps of gene activity from the brain and peripheral sensory neurons, they draw a detailed picture of “which” cells may carry the genetic signatures of chronic pain. Their results highlight two important players: glutamatergic neurons in the cortico-limbic circuit of the brain and C-fiber sensory neurons in the dorsal root ganglia (DRG) as key sites where pain risk is encoded (8).

## Connecting brain and body in chronic pain

The strength of this study lies in its rigorous methodology, which combines complementary gene-mapping approaches (stratified linkage disequilibrium score regression [S-LDSC] and multimarker analysis of genomic annotation [MAGMA]) and required both to converge on consistent results before declaring a cell type significant to chronic pain. This dual strategy minimizes false-positives that can arise from differing analytical assumptions. The brain’s role in chronic pain extends far beyond sensation — it involves how pain is remembered, anticipated, and emotionally processed. Toikumo and colleagues found that genetic risk for pain is concentrated in excitatory (glutamatergic) neurons within regions such as the prefrontal cortex, hippocampus, and amygdala (8). These regions form what is known as the cortico-limbic circuit, which shapes both the emotional and cognitive experience of pain (9–11). The findings refine earlier work from this group of collaborators that highlighted inhibitory (GABAergic) neurons in pain intensity (12), suggesting that the greater cellular resolution afforded by the current dataset (461 cell-type clusters) reveals a more predominant role for excitatory circuits (8). These discovery insights align powerfully with decades of neuroimaging and magnetic resonance spectroscopy (MRS) studies linking chronic pain to altered glutamatergic tone in these same brain areas (13, 14). By identifying the exact cell types involved within these circuits, Toikumo and colleagues add molecular precision to what brain imaging has long shown — the brain’s excitatory pathways sustain persistent pain.

Toikumo et al. showed that genetic risk also converges on C-fibers, the neurons in the DRG that detect noxious stimuli like heat or tissue damage (8). Certain subtypes of these neurons, including hPEP.PIEZOh (characterized by high PIEZO2 expression) and hAδ.LTMR (high expression of PIEZO2 and the neuronal RTK NTRK2), were associated with pain in specific body regions such as the joints and knees, respectively (8). While some of these associations did not survive correction for multiple comparisons, the pattern suggests that different pain sites may be shaped by genetic variation in distinct DRG neuron populations. Together, these findings paint a coherent picture: chronic pain risk is not scattered randomly across the body but focuses on 2 major circuits — excitatory brain neurons and peripheral neurons — that continuously communicate to construct the pain experience.

## The context of pain matters

A major advancement of this study was its use of transcriptomics data from patients who were actively experiencing pain, rather than relying solely on data from healthy individuals. Using single-cell RNA-Seq of cervical DRG samples from patients with acute and chronic pain, the authors found that genetic risk variants were particularly enriched in genes upregulated during chronic pain states, such as *GABBR1*, *NCAM1*, *EFNB2*, and *NRXN1* (8). This means that many of the genes carrying genetic risk are not just passively expressed; rather, they become activated in the context of ongoing pain. Interestingly, while the well-known sodium channel genes *SCN9A* (encoding Nav1.7) and *SCN10A* (Nav1.8) showed no enrichment, *SCN11A* (Nav1.9), which is critical in regulating DRG excitability, was specifically enriched in neuronal cell clusters, emerging as a key candidate (8). This does not mean that Nav1.7 and Nav1.8 are irrelevant for chronic pain; rather, their absence may reflect the broad, mixed-phenotype nature of current chronic pain GWAS datasets. Importantly, this study also highlights the growing importance of non-neuronal cells in pain susceptibility. GWAS signals were enriched not only in neurons, but also in astrocytes and oligodendrocyte precursor cells located in both the brain and spinal cord (8). These findings reinforce the idea that chronic pain is not solely a neuronal disorder: it arises from a dynamic communication between neurons, glial cells, and the immune system, which together shape pain persistence and recovery.

## Challenges and future directions

While the study offers a powerful framework for translational research, there are several important limitations that define the next generation of pain genomic studies. A primary challenge lies in phenotype heterogeneity. The pain assessment methods varied across the large biobanks used in the GWAS analysis by Toikumo et al. (8). This variability makes it harder to identify the shared biology underlying chronic pain as a single condition. Therefore, as a result, some genetic associations may reflect general pain sensitivity rather than specific conditions. Future studies should prioritize deep, harmonized phenotyping to identify mechanisms shared across different pain types.

**Figure 1:**
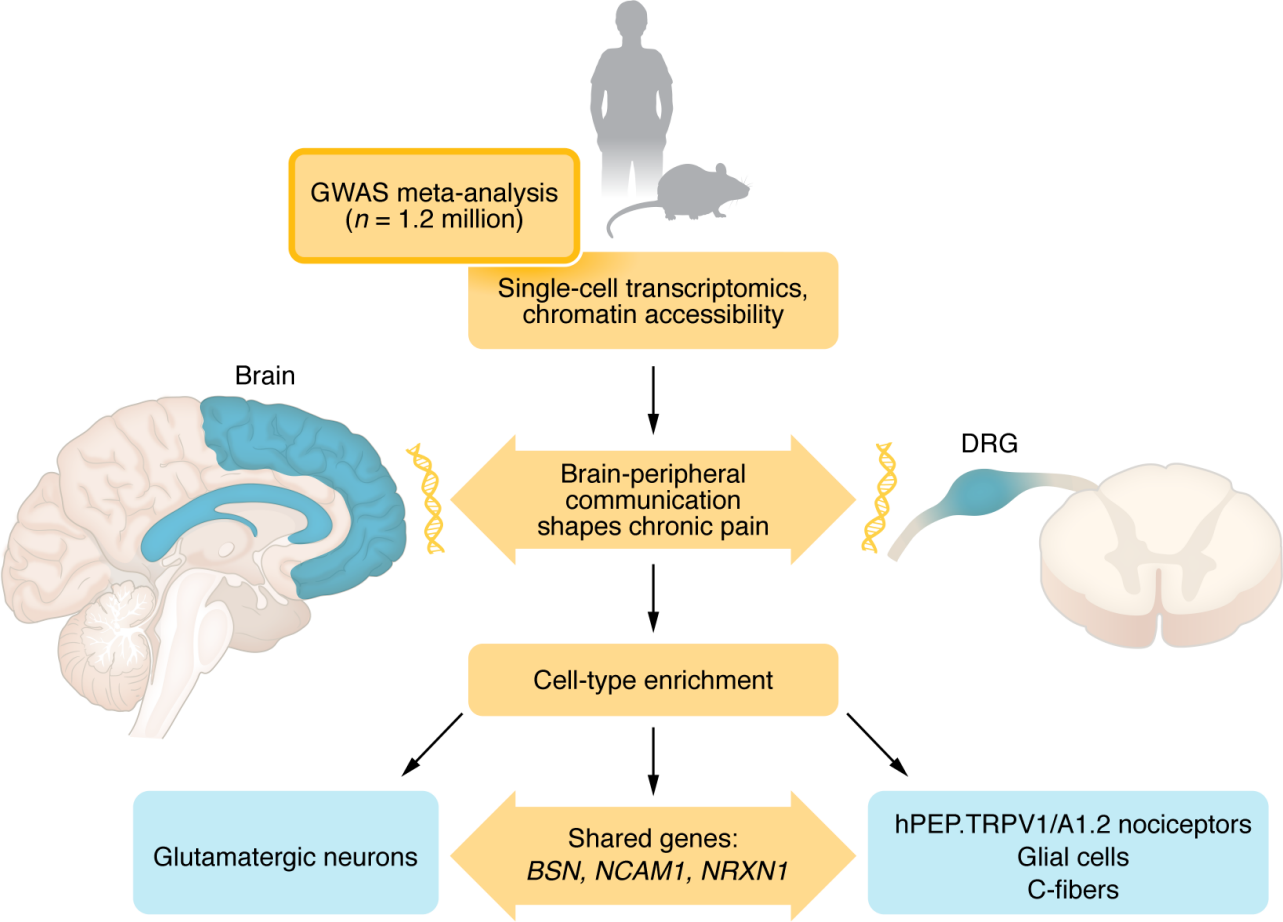
A genetic roadmap linking the brain and body in chronic pain. Toikumo et al. (8) combined genetic data from more than 1.2 million individuals with single-cell transcriptomics and chromatin accessibility analyses from the human brain and DRG. The integrated data were complemented by chromatin accessibility profiling in mouse spinal dorsal horn tissue. These analyses revealed that genetic variants are enriched in glutamatergic neurons of cortico-limbic circuits, C-fiber nociceptors (hPEP.TRPV1/A1.2), and glial cells. Shared genes such as *BSN*, *NCAM1*, and *NRXN1* connect central and peripheral pathways, showing how genetic susceptibility is distributed across an integrated network. This bidirectional communication between brain and periphery shapes both the emotional-cognitive and sensory aspects of chronic pain.

Another challenge is causality. Identifying a genetic variant linked to pain doesn’t necessarily mean that the gene causes pain — it may simply be associated with it. Many of the differences observed in genes differentially expressed could reflect the body’s response to pain rather than its origin. Proving causality will require direct experiments to confirm whether these genes cause pain susceptibility or simply represent downstream adaptations. Furthermore, the integration of data across species warrants cautious interpretation. The need to use mouse orthologs for spinal cord chromatin accessibility highlights the current lack of high-quality human spinal cord data. Although mouse analysis benefits from evolutionary conservation in many enhancers, species differences in circuitry and the gulf between subjective human pain experience and reflexive rodent assays require ongoing consideration. Toikumo et al. reported preliminary evidence of sex differences, noting a greater enrichment of pain-related brain cell types in males compared with females (8). These results should be interpreted carefully, given that most participants in the largest datasets were male individuals. Future studies achieving sex-balanced and ancestrally diverse genomic datasets will be crucial to fully understand the genetic landscape of pain. Together, these insights outline a multi-scale view of chronic pain — from genes to cells to circuits — that now guides how we will design the next generation of therapies (Figure 1).

## From discovery to therapy

By identifying the specific neurons and circuits where chronic pain risk resides, the study by Toikumo et al. offers a roadmap for precision medicine (8). The strong enrichment in cortical and limbic glutamatergic neurons suggests that analgesics capable of crossing the blood-brain barrier could be crucial for long-term relief, targeting the circuits that process pain rather than only the nerves that detect it. At the same time, identifying specific DRG nociceptor subtypes offers opportunities to fine-tune peripheral therapies, minimizing side effects by focusing only on the neurons relevant to particular pain types. Future drug discovery efforts could focus on the genes shared between central and peripheral circuits such as *BSN*, *NCAM1*, and *NRXN1*, which are involved in synaptic organization and communication. Targeting the genes that are activated during chronic, rather than acute, pain may be the key to developing treatments that truly modify the disease rather than temporarily masking its symptoms.

## Conclusion

The study by Toikumo and colleagues moves the field beyond identifying “where the pain genes are” to understanding “which cells” translate those genes into persistent pain (8). With their integrative analysis connecting population-scale genomics with cellular identity, the authors show that chronic pain risk converges on a network of excitatory neurons in the brain and sensory neurons in the periphery. For researchers and clinicians alike, this represents a new way to think about pain: not as a diffuse and mysterious experience, but as a condition with a definable cellular origin and a traceable genetic signature. This roadmap brings us closer to the long-sought goal of developing safe, nonaddictive, and effective therapies that address the root causes of chronic pain.

## Funding support

This work is the result of NIH funding, in whole or in part, and is subject to the NIH Public Access Policy. Through acceptance of this federal funding, the NIH has been given a right to make the work publicly available in PubMed Central.

- National Institute of Neurological Disorders and Stroke (NINDS), NIH (NS098772 and NS120663, to RK).
- PhRMA Foundation Postdoctoral Fellowship in Drug Discovery (no. 1335819, to EJRP).

## Version 1 12/15/2025

Electronic publication
